# Some Points in the Diagnosis of Hip Disease in Children

**Published:** 1891-09-19

**Authors:** 


### THE PRACTITIONER'S MIRROR AND RETROSPECT.

[*The Editor will be glad to receive offers of co-operation and contributions from members of the profession. All letters should be addressed to* THE EDITOR, THE LODGE, PORCHESTER SQUARE, LONDON, W.]

### SURGERY.

### SOME POINTS IN THE DIAGNOSIS OF HIP DISEASE IN CHILDREN.

We have lately seen several cases of hip joint disease which well illustrate its diagnosis ; and they all occurred in children. A few days ago we were asked to examine a young child on one of whose lower limbs extension had been applied for hip joint disease, but who was in greater pain after the apparatus was put on than before. On removing the weight the limb became very flexed ; indeed, the extension had not wholly overcome the flexion when it was working. Certainly there was great limitation in the movement at the hip, but it was a limitation of extension only ; as soon as we tried to overcome the flexion beyond the slightest possible extent, pain was at once caused, and the pelvis began to move with the femur, but on rotating the joint there was fairly free and painless movement, nor did jarring the joint surfaces together cause the slightest pain. The abdomen was considerably distended, and we could not make much out, but on dipping down internal to the anterior superior spine there was certainly a suspicious fulness in the iliac region on that side. There was no angular curvature in the spine, and no specially tender point there, but we think it most likely there is some caries of the anterior part of one or more vertebræ or of the ilium, causing a psoas abscess which has not yet descended into the thigh, and that the irritation of the psoas causes the painful contraction of the thigh. The iliac swelling seems too deep to be perityphlytic in origin, and there are no signs of sacro-iliac disease. We may have caries of the spine without excurvation, and we are disinclined to accept the statement that psoas abscess may sometimes be due to "inflammation of areolar or fascial structures," or that "acute idopathic abscess may occur in children." It seems to us much more likely that there is in these cases some very limited bone disease somewhere in the course of the muscle. We must remember what an extent of bone the psoas arises from. Abscess in any other muscle except traumatic or pyæmic is practically unknown. Only the other day we made a post-mortem on a case where a psoas abscess was tracking up towards the spine instead of down, but this was not from primary "irritation of fascial structures," but from disease in the pubes. In the case we have been describing the child was so lamed by the painful contraction of the thigh that the usual test of fixation or otherwise of the spine could not be applied.

We have notes of a very similar case seen nine years ago. The patient was older (13), but here again the first symptoms were lameness from great flexion of the thigh, accompanied, however, by a complaint of pain in the back, which the other child was too young to complain of if present. The flexion was extreme, and extension was not possible, but all other movements at the hip were free and painless. Here, also, there was a deep iliac, but no groin swelling, and no excuvation or tenderness of the spine. But the diagnosis was confirmed here by the presence of "much soft swelling and tenderness" on one side of the lumbar spine.

But with regard to the case first described, there is another point of great interest. The child is passing large quantities of pus and a little blood, but as the urine is acid, presumably not originating in the bladder, although no casts are seen. Abscess from diseased spine has been known to burst into the pelvis of the kidney, and so give rise to the presence of pus in the urine, and we are inclined to think this may be so here, for there is a complete absence of all urinary symptoms, and the amount of pus is sometimes nearly equal to the bulk of urine after standing, and presents a very curious appearance. First, at the bottom of the conical glass are a few dark clots, then a deep layer of pus, and then a fairly deep layer of red corpuscles, reminding one very much of the ornaments that used to be made of different layers of coloured sand from the Isle of Wight. Then, again, the inability to feel any renal swelling, or make out any renal tenderness, and the presence of the deep iliac swelling seem to us more in favour of spinal caries with psoas abscess than pyelitis, with a perinephritic abscess, causing irritation of the psoas muscle and flexion of the thigh, as we know it may do. If we can find any minute masses of bone in the pus in the urine the source of the disease will of course be proved.

We wish now to refer to another case illustrating the diagnosis of hip disease. A few weeks ago we were asked to see a child who was an inmate of a workhouse, and whose previous history was unknown. She limped ; one leg was much wasted and seemed much shortened, and these symptoms had suggested her removal to hospital for hip disease. But movement at the hip was too free and painless to justify the diagnosis, and although the muscles of the thigh were much wasted the wasting was general in the limb. Actual shortening, however, was present to a marked extent. Was it then a case of recovery from old hip disease in which growth at the upper epiphysis of the femur had been interfered with although destructive disease had not proceeded so far as to cause ankylosis. Measurement from the patella to the internal maleolus settled the question, for it disclosed the fact that the bones of the leg were atrophied as well as the thigh bone. Clearly the case was one of old infantile paralysis, and instead of being treated by prolonged rest, demanded massage and faradisation of the muscles.

We should like to call attention to some cases of primary disease in the pubic bone causing hip disease. A few months ago we saw a baby with an enormous abscess presenting behind the trochanter. There were no definite signs of spinal disease, and free rotation of the hip under chloroform did not produce grating. The abscess was opened, and was found to communicate with the joint, which, though freely open, was not rough ; and one's finger passed upwards over the edge of the pubes, the abscess cavity extending very much to the inner side of the thigh, internal to the hip joint. Looking at the fact that there was no bare surface in the hip joint, that the cavity extended up over the pubes, and that a minute sequestrum found in the pus had not probably come from the hip joint, we were inclined to think it was a psoas abscess, with secondary destruction of the joint, as sometimes occurs, for we have several times seen a psoas abscess point behind the trochanter. But the post-mortem showed the primary disease to be in the horizontal ramus of the pubes, where there was a cavity which would just have held the sequestrum found in the pus from the abscess. There certainly was a psoas abscess, but it was tracking from the pubes up to the spine, and did not reach the spine, nor could any disease of the vertebræ be discovered on very careful dissection. The joint disease was secondary, due to an abscess opening into the joint, but not a spinal psoas abscess.

